# Acetoin Catabolism and Acetylbutanediol Formation by *Bacillus pumilus* in a Chemically Defined Medium

**DOI:** 10.1371/journal.pone.0005627

**Published:** 2009-05-20

**Authors:** Zijun Xiao, Cuiqing Ma, Ping Xu, Jian R. Lu

**Affiliations:** 1 Center for Bioengineering & Biotechnology, China University of Petroleum, Qingdao, People's Republic of China; 2 State Key Laboratory of Microbial Technology, Shandong University, Jinan, People's Republic of China; 3 MOE Key Laboratory of Microbial Metabolism and School of Life Sciences and Biotechnology, Shanghai Jiao Tong University, Shanghai, People's Republic of China; 4 Biological Physics Group, School of Physics and Astronomy, The University of Manchester, Manchester, United Kingdom; Instituto Butantan, Brazil

## Abstract

**Background:**

Most low molecular diols are highly water-soluble, hygroscopic, and reactive with many organic compounds. In the past decades, microbial research to produce diols, e.g. 1,3-propanediol and 2,3-butanediol, were considerably expanded due to their versatile usages especially in polymer synthesis and as possible alternatives to fossil based feedstocks from the bioconversion of renewable natural resources. This study aimed to provide a new way for bacterial production of an acetylated diol, i.e. acetylbutanediol (ABD, 3,4-dihydroxy-3-methylpentan-2-one), by acetoin metabolism.

**Methodology/Principal Findings:**

When *Bacillus pumilus* ATCC 14884 was aerobically cultured in a chemically defined medium with acetoin as the sole carbon and energy source, ABD was produced and identified by gas chromatography – chemical ionization mass spectrometry and NMR spectroscopy.

**Conclusions/Significance:**

Although the key enzyme leading to ABD from acetoin has not been identified yet at this stage, this study proposed a new metabolic pathawy to produce ABD *in vivo* from using renewable resources – in this case acetoin, which could be reproduced from glucose in this study – making it the first facility in the world to prepare this new bio-based diol product.

## Introduction

Acetoin (3-hydroxy-2-butanone) is a volatile compound naturally existing in wine, honey, cocoa, butter, coffee, strawberry, garnet berry, etc [1]. It not only has multiple usages in foods, flavor, cosmetic, and chemical synthesis, but also works as one of the important primary metabolites like ethanol, acetate, and lactate, in various microorganisms. The excretion of acetoin, which can be diagnosed by the Voges Proskauer test and serves as a microbial classification marker, has its vital physiological implications to these microbes [2].

*Bacillus*, such as *Bacillus subtilis*, is one of the mostly studied Gram-positive bacterial species. The acetoin-excreting genus produces spores to resist unfavorable conditions such as UV radiation, *γ*-radiation, H_2_O_2_, desiccation, chemical disinfection, or starvation. *Bacillus pumilus*, although taxonomically close to *B. subtilis*, is something different. For example, it is incapable of the hydrolyzation of starch [3]. Some of *B. pumilus*, including both spores and vegetative cells, exhibit elevated resistant abilities compared to other *Bacillus* species [4]. The start of acetoin catabolism and sporulation signifies the two important stages of life in this bacterium. There have been many reports concerning acetoin metabolism in *B. subtilis*, but few are available in *B. pumilus*. However, as one of the best acetoin producers for possible desired industrial benefits [5], acetoin metabolisms in *B. pumilus* still remain unclear.

**Figure 1:**
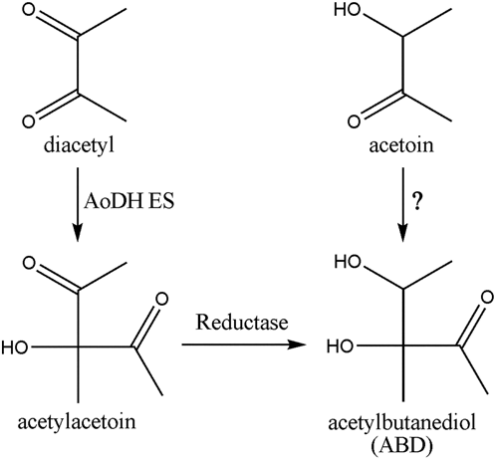
The molecular structure and proposed formation mechanisms of ABD. Left, acetylacetoin formation from diacetyl by the acetoin dehydrogenase enzyme system (AoDH ES) and its reduction to ABD [2]; Right, speculative one step direct ABD formation from acetoin (this study, the enzyme has not been identified).

Acetylbutanediol (ABD, with its formal IUPAC name of 3,4-dihydroxy-3-methylpentan-2-one) is an intermediate previously introduced in 2,3-butanediol cycle [6]. Although this cyclic route has been exclusively reviewed [2], the formation of ABD is widely recognized as the reduction product of acetylacetoin (Fig. 1).

Having the virtue of confirmatory ingredients and high reproductivity, chemically defined media (CDMs) have been widely used in the study of microbial metabolism. But there are seldom reports on the culture of *B. pumilus* in CDM. In this study, an ABD preparation method from a CDM containing acetoin as the sole carbon and energy source was realized by aerobic fermentation using *B. pumilus* ATCC 14884. A new formation mechanism for ABD by direct acetylation of acetoin was postulated.

## Results

### Acetoin as sole carbon source for ABD production

**Figure 2:**
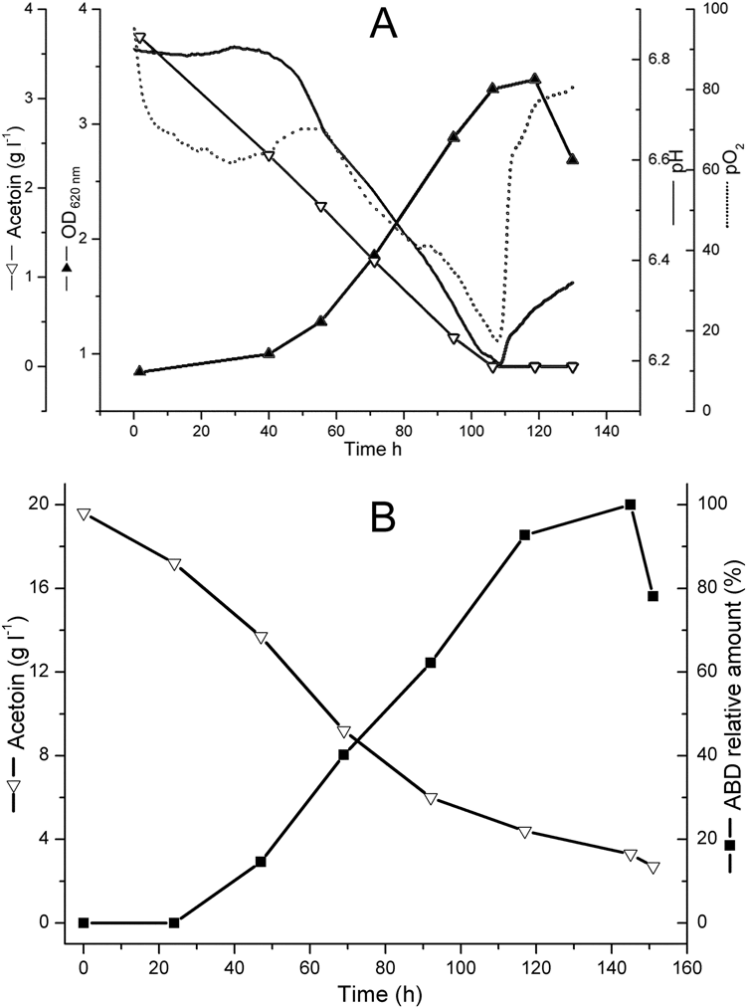
Pre-culture fermentation (A) and ABD production process (B) using acetoin as the sole carbon source in CDM by *B. pumilus* ATCC 14884. ABD conversion was presented in relative percentage.

Fig. 2A shows the time dependent growth curve of *B. pumilus* ATCC 14884 in the pre-culture fermentation using acetoin as the sole carbon and energy source. During the course of acetoin degradation, acetate was detected, and dissolved oxygen level (DO) and pH value both decreased sharply. The DO reduction could be explained by cell growth, reproduction, and thus elevated respiration. The pH reduction could be explained by the conversion of acetoin to acetate catalyzed by AoDH ES [2]. At 106.3 h, acetoin (initial concentration 3.7 g l^−1^) was exhausted and OD_620 nm_ grew to 3.3. After this point, acetate excreted into the culture medium was assimilated by the cells, resulting in the increase of pH. Under the stress of carbon shortage, the bacterium experienced the stationary phase and entered the death phase subseuqnetly.

For ABD production (Fig. 2B), 19.6 g l^−1^ acetoin was fed into the fermentation system at the end of the pre-culture course. Because the cells in the system became accustomed with acetoin as the sole carbon and energy source, in other words, the cells had synthesized all the enzymes indispensable for cell growth and reproduction using acetoin as the initial substrate, acetoin degradation was much faster than that in the pre-culture course. This difference is apparent from the rates of reduction of acetoin in Fig. 2A and B. ABD in the medium could not be detected at the first 24 h, as is apparent from the initial flat region of the ABD profile in Fig 2B, indicating the occurrence of the induction period in the process. Thereafter, the amount of ABD showed a steady increase, reached its peak region between 120 and 140 h and then started to decrease. The fermentation process was then stopped to harvest the broth for ABD extraction.

### ABD extraction and GC-MS analysis

**Figure 3:**
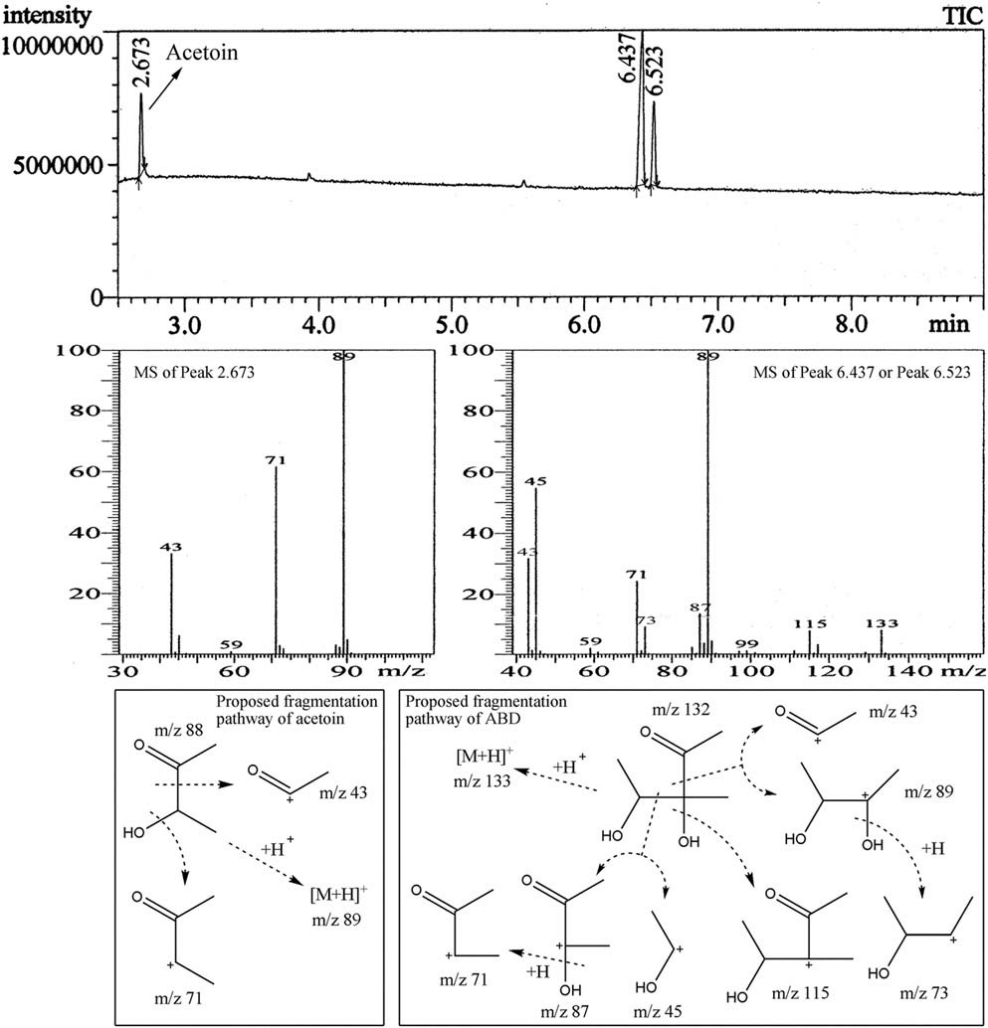
GC-CIMS of the ABD product. Up: total ion chromatogram (TIC) to show the occurrence of the 3 peaks; Middle: CIMS from the peaks with retention time of 2.673 min (left), 6.437 min and 6.523 min (right). The last two peaks share an entirely identical CIMS profile. Down: proposed CIMS fragmentation pathways of acetoin (left) and ABD (right).

By extraction and concentration, a thick colorless product (0.55 g) was recovered from 4.5 liter of fermentation broth. As described previously, product purity was determined using the Varian GC system equipped with a flame ionization detector (FID). Data based on the GC normalization method using FID showed that the components corresponding to those in the total ion chromatogram (TIC) (Fig. 3, up) were acetoin (Peak 2.673) 4%, ABD I (Peak 6.437) 68%, and ABD II (Peak 6.523) 23%, respectively. (For the introduction and identification of ABD I and ABD II, see below.)

As is well known, MS in electron impact ionization mode (EI mode) is one of the most powerful analysis methods for the identification of low molecular volatile organic compounds. However, ABD is a rather fragile molecule in EI mode (this information is based on the unpublished work from our previous study). In order to find the molecular ion peak of ABD, GC-MS in CI mode was performed (Fig. 3, middle) and the TIC was recorded (Fig. 3, up). The CIMS fragment ions of Peak 6.437 and Peak 6.523 were coincidently the same, indicating that they were isomers of the same compound. Although the quasi-molecular ion ([M + H]^+^, m/z 133) signal was weak, which agreed with the fragile MS characteristic of ABD, it is consistent with the molecular weight of ABD, i.e. 132. The other peaks of the CIMS fragment ions were assigned attemptedly consulting that of acetoin (Fig. 3, down left). The proposed fragmentation pathway for ABD (Fig. 3, down right) could perfectly fit the needs of the MS fragment peaks of Peak 6.437 and Peak 6.523. Therefore, Peak 6.437 and Peak 6.523 were both tentatively identified as ABD.

There are four types of ABD stereoisomers, i.e. 3*R*,4*R*-ABD, 3*S*,4*S*-ABD, 3*R*,4*S*-ABD, and 3*S*,4*R*-ABD. The former two and the later two constitute two pairs of enantiomers, which were herein referred to as type I ABD (ABD-I) and type II ABD (ABD-II), respectively. Generally speaking, it is almost impossible to separate a pair of enantiomers by non-chiral chromatography. Thererfore, it is reasonable to assume that ABD-I and ABD-II are both present, but the current analyses could not specify the number of ABD isomers in the ABD product.

### NMR identification of ABD

**Figure 4:**
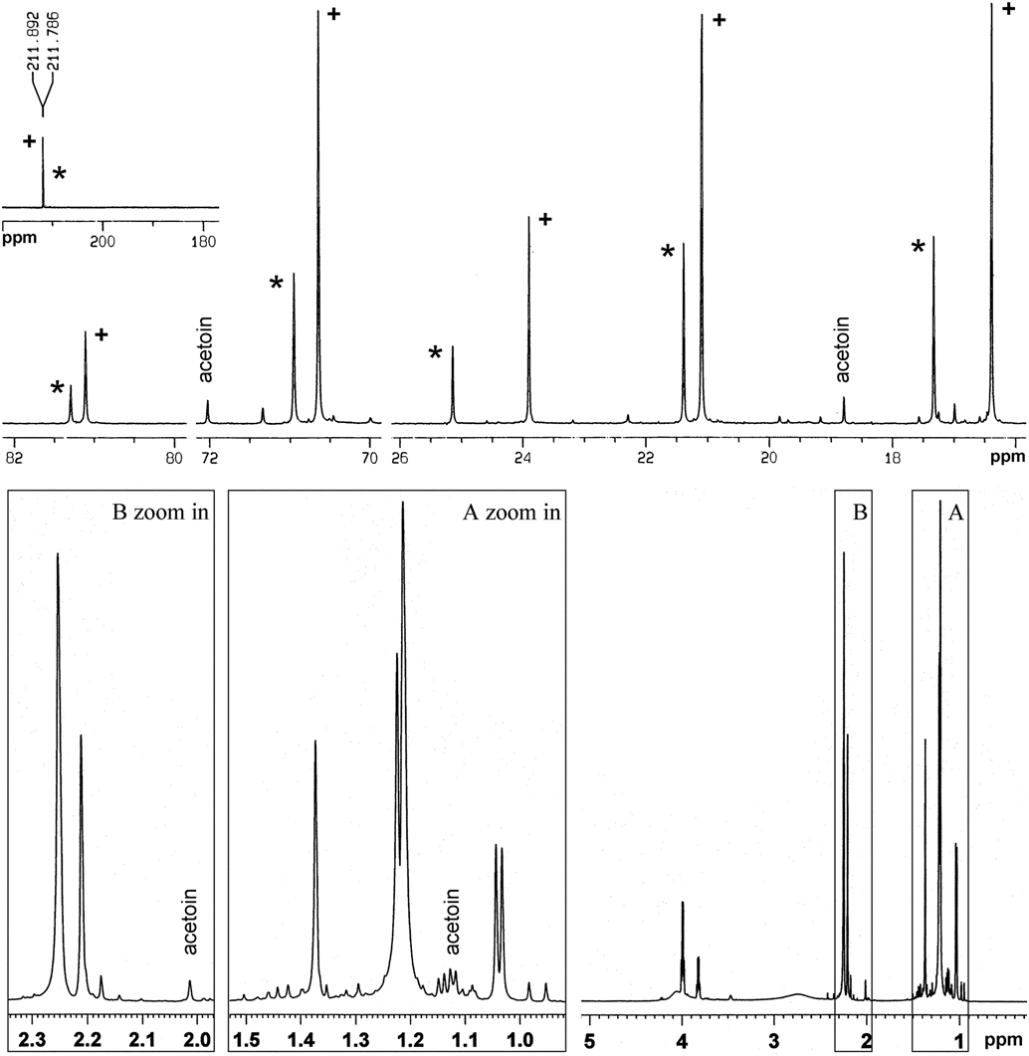
^13^C-NMR and ^1^H-NMR spectra of the ABD product. The sample was dissolved in CDCl_3_, and the chemical shifts were calibrated against TMS. The system was operated at 600 MHz for both ^13^C-NMR and ^1^H-NMR. Peaks with stars and pluses are the carbon signals of ABD-I and ABD-II, respectively.

The ABD product was further analysed by ^13^C-NMR and ^1^H-NMR using CDCl_3_ as the solvent and the resultant spectra are shown in Fig. 4. As shown in Fig. 1 or 3, one ABD molecule has six carbon atoms in different chemical environments. According to the GC-MS analysis, ABD-I and ABD-II were both present in the sample. As already indicated earlier, even if there were three or the maximal four ABD stereoisomers present in the sample, the enantiomeric one(s) could not be differentiated by NMR under the current experimental conditions in this study (non-chiral NMR, without the addition of chiral agents). Therefore, the two different enantiomeric types of ABD, i.e. ABD-I and ABD-II, should produce twelve different carbon signals corresponding to the carbon atoms in the twelve different chemical environments. The ^13^C-NMR spectrum clearly validated the eductions, as evident from Fig. 4 (up). The ^1^H-NMR spectrum also followed similar deductions, as evident from Fig. 4 (down).

The full NMR peak assignments are given in Table 1 using the information based on a previous study by Ui et al. [7]. Because *3R,4R*-ABD and *3S,4R*-ABD are a pair of diastereomers of ABD, they possess different chemical shifts corresponding to the same carbon atoms. The main impurity in the ABD product was identified to be acetoin, whose corresponding peaks in the NMR spectra are labeled in Fig. 4. Thus, the NMR analyses, as far as the presence of residual acetoin substrate in the ABD product is concerned, were also entirely consistent with the above GC-MS analysis.

**Table 1:** Chemical shifts of ABD-I, ABD-II, and peak assignments

Compounds	3*R*,4*R*-ABD^a^	ABD-I	3*S*,4*R*-ABD^a^	ABD-II
CH_3_COH	17.0	16.3879	17.9	17.3260
CH_3_C = O	21.6	21.0957	21.8	21.3837
CH_3_CHOH	23.8	23.8983	25.0	25.1409
CH_3_ CHOH	71.0	70.6469	71.2	70.9537
CH_3_ COH	81.1	81.1092	81.4	81.2943
CH_3_ C = O	211.4	211.892	211.0	211.786
3H, s, CH_3_COH	1.26	1.214	1.08	1.039
3H, s, CH_3_CHOH	1.28	1.224	1.44	1.373
3H, s, CH_3_C = O	2.30	2.252	2.25	2.211
1H, s, HCOH	4.02	3.997	3.87	3.827

a NMR assignments of 3*R*,4*R*-ABD and 3*S*,4*R*-ABD from Ui et al. [7], where data were acquired using CDCl_3_ as the solvent, TMS as the standard, at 125 MHz for ^13^C-NMR and 500 MHz for ^1^H-NMR, respectively.

The quantitative ratio of ABD-I to ABD-II in Fig. 4 was also consistent with that of Peak 6.437 to Peak 6.523 in the TIC of Fig. 3. However, this situation did not hold concerning the ratio of ABD-I (or ABD-II) to acetoin in Fig. 4 when compared with those of Peak 6.437 (or Peak 6.523) to Peak 2.673 (acetoin) in Fig. 3. Mass discrimination effects would account for the difference. The instrument GCMS-QP2010 (Shimadzu, Japan) used in this study was a quadrupole based GC-MS system. When a TIC was obtained in such a system, mass discrimination (ions at higher masses appear less) was a very common phenomenon [8]. This discrimination probably resulted from a decreased transmission of light ions through the quadrupole ion guide and aperture optics [9].

Combining all the results of the GC-MS and NMR analyses, Peak 6.437 and Peak 6.523, i.e. the main ingredients in the ABD product, were proved to be ABD-I and ABD-II, respectively.

## Discussion

We show in this study that when *B. pumilus* ATCC 14884 was aerobically cultured in CDM using acetoin as the sole carbon source, ABD was produced. The product was identified by both GC-MS and NMR. To our best knowledge, this is the first experimental report on ABD formation from acetoin catabolism in *B. pumilus*. ATCC 7061, the type strain of *B. pumilus*, was also found to be capable of acetoin catabolism and ABD formation in our previous work (results not yet reported). Therefore, this phenomenon could be a common characteristic of the *B. pumilus* species.

Although not very fast, *B. pumilus* ATCC 14884 can grow not only in the CDM as described previously, but also in the different CDM as described by Sergeant and co-workers [10]. We did not choose their medium because it contained glutamic acid, which might serve as a second carbon source. For better ABD fermentation, pre-culture using acetoin as the sole and initial substrate was necessary for the cells to synthesize the enzymes required for acetoin catabolism and cell reproduction when the cultivation was changed from a nutritious medium to a harsh CDM.

AoDH ES is the key enzyme system in charge of acetoin catabolism in bacteria [2]. During acetoin catabolism, ABD was previously suggested to be a byproduct of AoDH ES from diacetyl with acetylacetoin serving as the intermediate (Fig. 1, left). In this study, however, no diacetyl was detected in the substrate acetoin, whose purity was more than 96.0% by GC analysis. Water was the main impurity in the acetoin used and our analysis indicated that it occupied more than 90% of the total impurities. The reaction from acetoin to diacetyl *in vivo* in bacteria has been widely accepted to be unrealizable [2]. In addition, diacetyl was not detected during the pre-culture course and the ABD fermentation process. Given that acetoin and diacetyl as well as ABD and acetylacetoin share similar molecular structures (Fig. 1), respectively, we postulate that ABD was a direct transformation product of acetoin, probably catalyzed by AoDH ES. However, it must be stated that the assumption that AoDH ES is the key enzyme to ABD formation from acetoin is merely speculative at this stage. In this way, AoDH ES would be a bifunctional biocatalyst: dominant catalysis of cleaving acetoin into C_2_ carbon units (i.e. acetyl-CoA and acetaldehyde) [11] and auxiliary catalysis of combining acetoin into C_6_ carbon units (i.e. ABD, in this study). The unusual enzymatic activities of AoDH ES seem to present a paradox and they, including the biological meaning of ABD formation *in vivo*, need further scientific scrutiny. However as a matter of fact, bifunctional and moonlighting enzymes are quite common in living systems [12], supporting the idea that bacteria use multifunctional enzymatic activities to achieve multitasking cellular needs.

Containing two hydroxyl groups in each molecule, low molecular diols contribute to high water solubility, hygroscopicity and reactivity with many organic compounds. In the past decades, microbial research to produce 1,3-propanediol [13] and 2,3-butanediol [14]–[15] were considerably expanded due to the two compounds' versatile usages especially in polymer synthesis and as possible alternatives to fossil based feedstocks from the bioconversion of renewable natural resources. Thinking about the similar molecular structure to 2,3-butanediol and acetoin, ABD would be very useful in multiple areas, e.g., as a monomer of synthetic polymers and resins, an additive in foods, flavor and cosmetic. Having an acetyl group and two chiral centers, ABD was expected to offer particular meaningful properties in polycondensate preparation or in asymmetric chemistry. As acetoin, the substrate for ABD production, could be reproduced from glucose in this study, the ABD preparation method reported here was also a “green” process.

The new biosynthetic pathway as discovered in this work may offer a useful route towards future production of ABD, because the amount of ABD in the broth is easily detectable and the fermentation process could be easily optimized. The extraction of ABD is also easy to undertake. If the overall ABD extraction yield was ∼ 50%, the highest ABD level in the medium could have already reached 0.7 g l^−1^. There is hence possible horizon for testing the potential for enhancement in ABD level in the reaction medium. However, for better resolving all the possible biotechnical hurdles in future, a detailed study on one step acetoin to ABD transformation must be conducted with purified enzymes to reveal the reaction mechanism in bacteria.

## Materials and Methods

### Chemicals

Natural acetoin (GC≥96.0%), a fermentative product from glucose, was a kind gift from Shanghai Apple Flavor & Fragrance Co., Ltd. (China). All other chemicals used in this investigation were of analytical grade bought from Sigma-Aldrich and used as supplied.

### Microorganism and cultivation

The strain used in this study was *B. pumilus* ATCC 14884, which is widely used in the assay of antibiotics [16]–[17] and as a sterilization control [18]. The seed culture, prepared as mentioned previously [1], was washed with sterilized saline before inoculated into fermentation medium, which was a modified CDM from Juni and Heym [6] containing: 2.72 g l^−1^ KH_2_PO_4_, 10.75 g l^−1^ Na_2_HPO_4_, 0.10 g l^−1^ MgSO_4_·7H_2_O, 2.00 g l^−1^ NH_4_Cl, 0.01 g l^−1^ CaCl_2_, 0.01 g l^−1^ vitamin B1, 0.30 mg l^−1^ FeSO_4_·7H_2_O, 0.01 mg l^−1^ MnSO_4_·H_2_O, 3.00 µg l^−1^ biotin, and variable amounts of acetoin as the sole carbon source.

### ABD fermentation and extraction

Pre-culture and ABD fermentation experiments were conducted using the above CDM in a 5 L fermenter (BIOSTAT B, B. Braun Biotech International GmbH, Germany). The fermenter was operated at 37°C, with stirring at 300 rpm, air flow at 1.0 vvm, initial pH set at 6.8. The pH was then uncontrolled during cultivation. The final fermentation broth containing ABD was extracted with chloroform and the organic phase was collected and concentrated under nitrogen protection.

### Analytical methods

During cultivation, cell growth was measured spectrophotometrically at the wavelength of 620 nm (OD_620 nm_). The culture broth was withdrawn periodically and the products were extracted with chloroform and analyzed using a GC system (Varian CP-3380, USA). Acetoin was subsequently quantitated as previously described [1]. Qualitative analysis for unknown peaks was performed using GC-chemical ionization mass spectrometry (GCMS-QP2010, Shimadzu, Japan) equipped with a 30 m DB-5MS column (0.25 mm inside diameter, 0.25 µm film thickness) (J & W Scientific, USA). The injector temperature was set at 280°C; the column oven was kept constant at 40°C for 3 min, and then programmed to 240°C with a temperature increase of 20°C min^−1^ and maintained at 240°C for 5 min. The temperature of the interface and the chemical ionization source were set at 220°C and 200°C, respectively. Positive chemical ionization was applied and the mass spectra were generated in the scan range of 35–500 amu. The concentrated ABD sample was dissolved in CDCl_3_ and analyzed by a Bruker AVANCE 600 FT-NMR spectrometer (Switzerland) equipped with CryoProbe TM operated at 600 MHz for both ^13^C-NMR and ^1^H-NMR. Chemical shifts were calibrated against tetramethylsilane (TMS).
